# ﻿Two new species of *Macropelopia* (Diptera, Chironomidae) from Oriental China, delineated with morphology and COI sequences

**DOI:** 10.3897/zookeys.1210.127183

**Published:** 2024-08-27

**Authors:** Xiangliang Fang, Zigang Xu, Yuanyuan Yao, Yue Fu

**Affiliations:** 1 College of Biology and Agricultural Resources, Huanggang Normal University, Huanggang City, Hubei, 438000, China Huanggang Normal University Huanggang City China; 2 College of Agriculture, Yangtze University, Hubei, 434022, China Yangtze University Hubei China; 3 Tianjin Natural History Museum, Tianjin City, 300061, China Tianjin Natural History Museum Tianjin City China

**Keywords:** DNA barcoding, *Macropelopia*, morphology, non-biting midge, taxonomy

## Abstract

Two new species, Macropelopia (Macropelopia) excavata Xu & Fu, **sp. nov.** and Macropelopia (Macropelopia) quadrimacula Xu & Fu, **sp. nov.**, are described as male adults. A key to identify the males of *Macropelopia* from China is provided. Furthermore, in order to ascertain the genetic distance between these species and their morphological characteristics, mitochondrial cytochrome *c* oxidase subunit I gene sequences were uploaded to the National Center for Biotechnology Information. These COI sequences were then utilized to infer the relationships between the species, employing the neighbor-joining method.

## ﻿Introduction

Thienemann and Kieffer (1916) established the genus *Macropelopia*, with *Isoplastusbimaculatus* Kieffer as the type species. *Macropelopia* is classified in the tribe *Macropelopiini* and is further divided into two subgenera: *Bethbilbeckia* Fittkau et Murray and *Macropelopia* s. str. (Cranston and Epler 2013). Previous studies described a total of 18 species within the subgenus Macropelopia s. str. and three species in the subgenus Bethbilbeckia. The main difference between the two subgenera in the adult stage is that *Macropelopia* s. str. has a tibial comb, while Bethbilbeckia does not. Among these, 11 species of the subgenus Macropelopia are found in the Palearctic region (Kieffer 1912, 1916; Tokunaga 1937; Fittkau 1962; Lencioni and Marziali 2005), one in the New North region (Roback 1971), two in the Oriental region (Tang and Niitsuma 2020; Hazra and Chaudhuri 2001), three in the Neotropical region (Andersen 2018; Silva and Pinho 2020; Dantas et al. 2023), and one in the African region (Freeman 1955).

According to Wang (2000), only one species of *Macropelopia*, *M.nebulosa* (Meigen), has been described based on adult, and four other species have been recorded based on larvae. Wang et al. (2011) recorded three additional species of *Macropelopia*, namely *M.galbina* Wang, Cheng & Wang, and *M.grandivolsella* Wang, Cheng & Wang which were both found in Hubei Province, and *M.rotunda* Wang, Cheng & Wang, which was discovered in Fujian Province. However, recent examinations conducted by Tang and Niitsuma (2020) have resulted in significant taxonomic revisions. The species *M.grandivolsella* has been synonymized with *Macropelopiaparanebulosa* Fittkau, while *Macropelopiarotunda* is now referred to as *M.kibunensis* (Tokunaga), with these two species considered synonymous. Additionally, *M.galbina* was transferred to the genus *Brundiniella*. Furthermore, Tang and Niitsuma (2020) have described a previously unknown species, Macropelopia (Macropelopia) pergrandis, originating from Yunnan Province.

In this study, we report the discovery of two new species within the subgenusMacropelopia s. str., sourced from the remarkable Dabie Mountain National Nature Reserve situated in Hubei Province. The discovery holds significant importance as it contributes to the diversity of the genus. In addition, we have also assembled a key for distinguishing and identifying the known adult males of the subgenus in China. Moreover, we have conducted an analysis utilizing the mitochondrial cytochrome *c* oxidase subunit I (COI) gene to infer genetic distance and determine the differences between the species within the genus *Macropelopia*,further enhancing our understanding of their morphological characteristics.

## ﻿Material and methods

Specimens were collected using the light trap induction method and preserved in 85% alcohol. Subsequently, they were sent to the laboratory for preliminary species identification and assigned individual numbers under a microscope. Images of slide mounts were obtained using a Nexcope NE930 compound microscope equipped with Capture 2.1 software. Genomic DNA was extracted from the thorax and legs of the specimens using the Qiagen DNA Blood & Tissue Kit. PCR amplification of the COI gene was performed following the primers and temperature regimes described by Folmer et al. (1994). After DNA extraction, the transparent exoskeleton was rinsed with 96% ethanol and mounted in Euparal on microscope slides, along with the corresponding antennae, head, wings, and legs, following the protocol outlined by Sæther (1969). Morphological nomenclature adheres to Sæther (1980), and measurements include the minimum, maximum, and average values for at least three specimens. All specimens are currently housed at the College of Biology and Agricultural Resources, Huanggang Normal University, China. Evolutionary analyses were conducted using MEGA 11.

The main abbreviations and corresponding English terms used in this study (with the value of ‘*N*’ representing the number of measured specimens mentioned in the text) are as follows: TL, total length; WL, total wing length; Pfe, length of the forefoot; VR, Venarum ratio = length of Cu / length of M; Cu_1_ cubitus 1; AR, Antennal ratio = length of ultimate flagellomere / combined lengths of flagellomeres one to penultimate; Fe, femur; ta_1_–ta_5_, tarsomeres ta_1_–ta_5_; LR, ta(basal segment) / ti; BV, Fe + ti +ta_1_ / ta_2_ + ta_3_ + ta_4_ + ta_5_; SV, Fe + ti / ta_1_; R, Radius; R_1_, Radius 1; R_4+5_, Radius four and five; BR, largest bristle/width of ta_1_ about 1/3 from distal end; HR, length of gonocoxite / length of gonostylus; HV, total length (TL) / length of gonostylus × 10.

## ﻿Taxonomy

### ﻿Genus Macropelopia

Taxon classificationAnimaliaDipteraChironomidae

Thienemann

3C8CEB72-D75F-5181-A695-2152D49F734B

Macropelopia Thienemann in Thienemann & Kieffer, 1916: 497. Fittkau 1962: 102; Roback 1971: 87, 1978: 159; Fittkau and Murray 1986: 50; Murray and Fittkau 1989: 61; Epler 2001: 4.53; Niitsuma et al. 2004: 44; Cranston and Epler 2013: 53; Silva and Pinho 2020: 575; Tang and Niitsuma 2020.
Bethbilbeckia Fittkau et Murray, 1988: 253; Murray and Fittkau 1989: 46; Epler 2001: 4.29.

#### Type species.

*Isoplastusbimaculatus* Kieffer [=*Tanypusnebulosus* Meigen] by original designation.

### ﻿ Macropelopia excavata

Taxon classificationAnimaliaDipteraChironomidae

Xu & Fu sp. nov.

068E7F1A-876C-5BAB-BE97-DA113946C045

https://zoobank.org/2AA64D33-4F08-444C-AC77-FA080DBFBCAF

Fig. 1A–K

#### Type material.

***Holotype*** male (HNU: Cdbs60), China: Hubei Province, Huanggang City, Yingshan County, Dabie Mountain, Longtan Gorge, light trap, 31.0867°N, 115.8138°E, 486.71 m a. s. l., 7. IX. 2022, Zigang Xu.

#### Etymology.

The new species is named “*excavata*” derived from the Latin term “*excavatus*”, meaning concave, which aptly describes the inward concave shape of tergite IX’s posterior edge.

#### Diagnostic characters.

The distinguishing characteristics of this new species are the presence of two prominent longitudinal thick spots positioned in the middle of tergites II to IV, and the wing with brown markings on the distal end of Cu_1_ and basal part of cell an. Additionally, the tergites from V to IX display a distinctive brown hue, adding to their identification. The posterior edge of tergite IX is concave in shape, and the anal point is absent. The gonostylus is prominently curved at a right angle.

#### Description.

Adult male (*N* = 1)

Total length 4.69 mm, thorax length 1.45 mm, wing length 2.66 mm, TL/WL 1.76, WL/Pfe 2.19.

**Figure 1.**
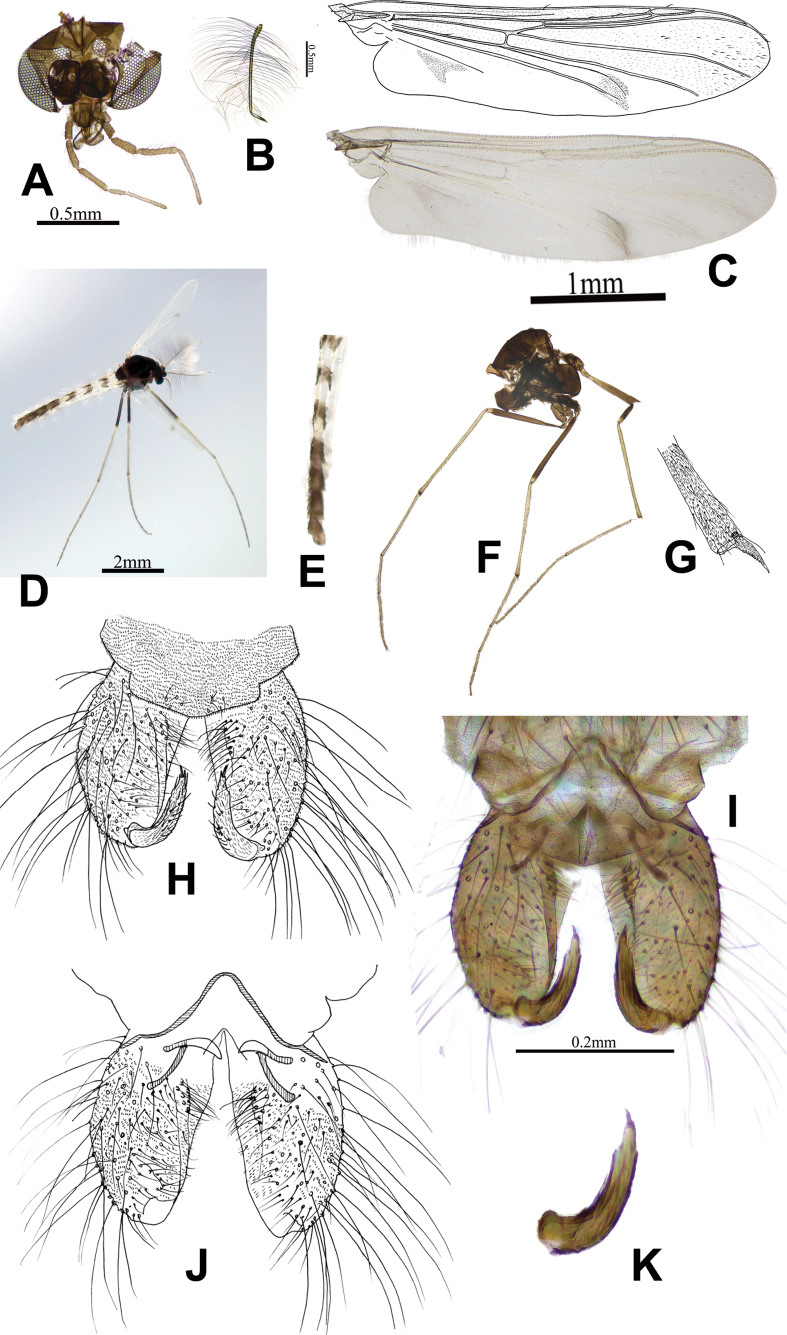
Macropelopia (Macropelopia) excavata Xu & Fu, sp. nov., male imago **A** head **B** antenna **C** wing **D** whole body of male adult **E** spotted shape of the tergites **F** thorax **G** fore tibial apex **H** hypopygium, dorsal view **I** photo of hypopygium, dorsal view **J** hypopygium, ventral view **K** gonostylus.

***Coloration*** (Fig. 1D). The head and thorax are uniformly dark brown. The femur of the legs is also dark brown, while the remaining parts of the legs are yellow. The wings exhibit two significant gray spots positioned near the Cu_1_ and An veins. There is a longitudinal color spot present in the middle of tergites II to IV, the tergites V to IX and hypopygium are all brown in coloration.

***Head*** (Fig. 1A). Antenna (Fig. 1B) with 13 flagellomeres, with an antennal ratio (AR) measuring 2.24. The flagellomeres taper towards their ends and ultimate flagellomere with a single apical seta. Temporal setae 24, comprising 8 inner verticals, 12 outer verticals, and 16 postorbitals. Tentorial length 234 µm, width 43 µm. Stipes length 192 µm, width 82 µm. Clypeus with 16 setae. Length of palpomeres (in µm): 69; 100; 161; 210; 342. Length ratio of palpomeres 5/3: 2.13.

***Thorax*** (Fig. 1F). Antepronotals with 9 setae, the acrostichals and dorsocentrals arranged in multiple columns, acrostichals with 41 setae, dorsocentrals with 62 setae. Postnotals with 6 setae, humerals with 8 setae, prealars with 32 setae. Scutal tubercle present and obvious, scutellars with 32 setae.

***Wing*** (Fig. 1C). The wing membrane area hairy, distinctive gray spots present near Cu_1_ and An veins, the arculus hook-like shape, and the anal lobe mainly developed. VR: 0.91, brachiolum with 3 setae, veins with many setae, squama with 34 setae. Costal extension 128 µm.

***Legs*.** The fore tibia possesses a single spur measuring 85 µm in length and features 15 side teeth. The width of the fore tibia at its apex is 84 µm, and the fore tibial comb consists of 5 setae (Fig. 1G). Two spurs of mid tibia are observed, measuring 87 µm and 51 µm long, bearing 16 and 11 lateral teeth, respectively, and has a width at the apex of 73 µm. The hind tibia exhibits two spurs, measuring 75 µm and 45 µm, with 18 and 12 lateral teeth, respectively. The hind tibial comb composed of 10 setae, with the longest seta 73 µm and the shortest 45 µm. The width of the hind tibia at its apex is recorded as 80 µm. The lengths and proportions of each leg are shown in Table 1.

**Table 1.** Lengths (in µm) and proportions of leg segments of male *M.excavata* Xu & Fu, sp. nov. (*N* = 1).

	fe	ti	ta_1_	ta_2_	ta_3_	ta_4_
P_1_	1215	1502	1168	600	428	293
P_2_	1295	1461	886	428	317	222
P_3_	1193	1654	1144	592	414	267
	**ta_5_**	**LR**	**BV**	**SV**	**BR**	
P_1_	208	0.78	2.54	2.32	3.96	
P_2_	164	0.61	3.23	3.11	4.45	
P_3_	184	0.69	2.74	2.49	4.56	

***Hypopygium*** (Fig. 1G, H). The anal point absent. The phallapodeme, although short, measures at a clearly discernible length of 69 µm. The gonocoxite showcases a cylindrical shape and spans 206 µm in length. The gonostylus (Fig. 1K) is 125 µm long and exhibits a curved inward shape at approximately two-thirds of its length. It displays protrusions on both the inside and outside, gradually narrowing towards the tip. The megaseta is 16 µm long. The inferior volsella unconspicuous, along the inside of gonocoxite, and contains concentrated long inner microhairs. HR: 1.64, HV: 3.74.

#### Remarks.

This new species is similar to *M.kibunensis* (Tokunaga) because their anal point is absent and the inferior volella is undeveloped, but can be distinguished by AR 2.24, wing with brown markings on distal end of Cu_1_ and basal part of cell an, otherwise unmarked, and a concave rear edge line of tergite IX, while AR 1.7–1.9, wing with brown markings on distal end of Cu_1_, M_3+4_ and M_1+2_. The pairwise distance based on the COI sequence of *M.kibunensis* and *M.excavata* sp. nov. is 0.105–0.107, further distinguishing them from each other.

#### Distribution.

Hubei Province, Oriental China.

### ﻿ Macropelopia quadrimacula

Taxon classificationAnimaliaDipteraChironomidae

Xu & Fu sp. nov.

BABD1E7E-6C04-50DB-8D4F-4207186D5AD0

https://zoobank.org/13C57663-77BF-4852-988D-F05C38B51B33

Fig. 2A–K

#### Type material.

***Holotype***, male (HNU: Cdbs7602), China: Hubei Province, Huanggang City, Yingshan County, Dabie Mountain, Longtan Gorge, 31.0867°N, 115.8138°E, 486.71 m a. s. l., 8. VI. 2022, light trap, leg. Zigang Xu. ***Paratypes***: 3 males (HNU: Cdbs7601, Cdbs7603, Cdbs7604), same as *holotype*; 3 males (HNU: Cdbs8901, Cdbs8902, Cdbs8903), Hubei Province, Huanggang City, Yingshan County, Dabie Mountain, Wujiashan National Forest Park, 31.1047°N, 115.7913°E, 931.91 m a. s. l., 9. VI. 2022, light trap, leg. Zigang Xu.

#### Etymology.

The name of this new species is derived from the Latin words “*quartri*” and “*macula*”, meaning “*four*” and “*spot*”, “*stain*” or “*mark*”, respectively. The name specifically pertains to the presence of four distinctive black spots found on the tergites of this species.

#### Diagnostic characters.

This species has two short longitudinal striped spots on each side of tergites II to V, as well as two elliptical spots in the center. Wing with brown markings on apical of Cu1, M3+4 and basal part of cell an. Additionally, tergite IX with a triangular anal point beyond the margin of tergite IX. Lastly, the gonostylus is curved inward at two-thirds of its length, and the apex is markedly tapered.

#### Description.

Adult males (*N* = 7)

Total length: 4.49–5.55, 5.02 mm, Wing length 2.72–3.27, 2.99 mm, TL/WL 1.65–1.76, 1.71, WL/Pfe 2.08–2.59, 2.34.

**Figure 2.**
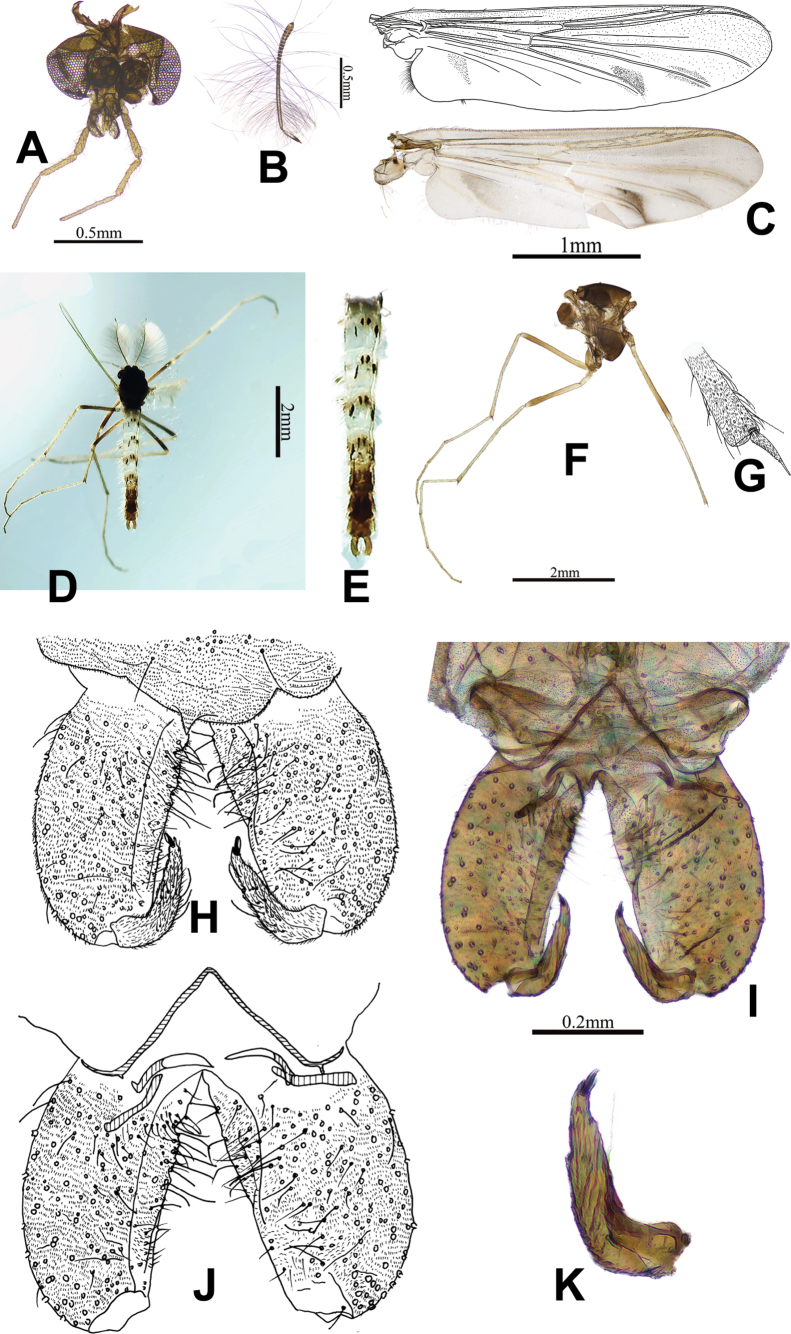
Macropelopia (Macropelopia) quadrimacula Xu & Fu, sp. nov., male imago **A** head **B** antenna **C** wing **D** whole body of male adult **E** spotted shape of the tergites **F** thorax **G** fore tibial apex **H** hypopygium, dorsal view **I** photo of hypopygium, ventral view **J** hypopygium, ventral view **K** gonostylus.

***Coloration*** (Fig. 2D). The head and thorax of this species are uniformly brown. The femurs of all legs are also brown, while the other sections display a yellow coloration. The wings are adorned with various color spots. Notably, there is a longitudinal color band on both sides of tergites II to V, accompanied by two elliptical spots at the center. Tergites VI to VII exhibit a dark brown hue, while tergites VIII to IX and the hypopygium are brown in color.

***Head*** (Fig. 2A). Antenna with 13 flagellomeres (Fig. 2B), AR: 2.01–2.05, 2.03, The end of flagellomere narrow, with approximately one-third of the end easily foldable, and the ultimate flagellomere possessing a single apical seta. Temporal setae 20–22, 21. The inner verticals and outer verticals are arranged in two or three columns, with 4–8, 6 inner verticals, 10–16, 13 outer verticals, and 12–18, 15 postorbitals. Tentorial length 250–258, 254 µm, width 36–43, 40 µm. Stipes length 271–303, 287 µm, width 62–95, 78 µm. Clypeus with 16–18, 17 setae. Length of palpomeres (in µm): 67–78, 72; 91–105, 98; 173–190, 182; 241–279, 260; 334–378, 356. Length ratio of palpomeres 5/3: 1.93–1.99, 1.96.

***Thorax*** (Fig. 2F). Thorax length 1.36–1.73, 1.55 mm, antepronotals with 12–12, 12 setae. The acrostichals and dorsocentrals are arranged in multiple columns, acrostichals with 40–60, 50 setae, dorsocentrals with 60–80, 70 setae. Anepisternals with 2–3, 3 setae, postnotals with 4–6, 5 setae, humerals with 8–14, 11 setae, prealars with 22–26, 24 setae. Scutal tubercle was present and obvious, scutellars with 32–40, 36 setae.

***Wing*** (Fig. 2C). The wing membrane area is hairy, Cu_1_ and An with obvious gray spots around the veins, arculus hook-shaped, and anal lobes particularly developed. VR: 0.91–0.96, 0.94, brachiolum with 3 setae, R_1_ with 30–40, 35 setae, R with 40–60, 50 setae, R_4+5_ with 50–60, 55 setae, squama with 54–60, 57 setae. Costal extension 157–160, 158 µm.

***Legs*.** The fore tibia possesses a single spur measuring 85–112, 98 µm in length and features 19–20, 20 side teeth. The width of the fore tibia at its apex is 79–86, 82 µm, and fore tibial comb consists of 5–6 setae (Fig. 2G). Two spurs on mid tibia are observed, measuring 87–95, 91 µm and 50–56, 53 µm long, bearing 18–20, 19 and 11–16, 14 lateral teeth, respectively, and has a width at the apex of 75–81, 78 µm. The hind tibia exhibits two spurs, measuring 88–96, 92 µm and 49–53, 51 µm, with 19–21, 20 and 15–17, 16 lateral teeth, respectively. The hind tibial comb is composed of 13–15,14 setae, with the longest comb seta 73–81, 77 µm and the shortest 40 –46, 43 µm. The width of the hind tibia at its apex is recorded as 76–86, 81 µm. The lengths and proportions of each leg are shown in Table 2.

**Table 2.** Lengths (in µm) and proportions of leg segments of male *M.quadrimacula* Xu & Fu, sp. nov. (*N* = 6).

	fe	ti	ta_1_	ta_2_	ta_3_	ta_4_
P_1_	1260–1321, 1291	1606–1803, 1705	1195–1346, 1271	628–712, 670	455–517, 486	300–342, 321
P_2_	1342–1526, 1434	1546–1732, 1639	944–1086, 1015	474–533, 504	343–394, 368	229–257, 243
P_3_	1285–1338, 1311	1795–1935, 1865	1167–1355, 1261	637–723, 680	455–498, 477	285–319, 302
	**ta_5_**	**LR**	**BV**	**SV**	**BR**	
P_1_	192–215, 203	0.73–0.75, 0.74	2.47–2.61, 2.54	2.28–2.46, 2.37	2.57–3.84, 3.21	
P_2_	165–184, 175	0.61–0.63, 0.62	3.16–3.21, 3.19	3.01–3.11, 3.06	2.56–4.37, 3.47	
P_3_	176–204, 190	0.63–0.71, 0.67	2.65–2.83, 2.74	2.42–2.67, 2.55	2.84–3.47, 3.16	

***Hypopygium*** (Fig. 2H–K). The anal point is small, forming an obtuse triangle. Phallapodeme short and obvious, 72–81, 77 µm long. Gonocoxite cylindrical shape, 253–265, 259 µm long. Gonostylus (Fig. 2K) 126–146, 136 µm long, curved inward at two-thirds of its length and possessing protrusions both on the inner and outer sides, the inner protrusion was located at a quarter of the apex of the gonostylus, while the outer protrusion was located halfway along the apex. Tergite IX with 16–20, 18 setae, megaseta 16–17 µm long. Inferior volsella small and protuberant. HR: 1.91–2.09, 2.00. HV: 3.56–3.94, 3.75.

#### Remarks.

This new species can be identified by the presence of two short longitudinal color bands on the sides of tergites II to V, along with two elliptical spots in the middle. These distinctive characteristics set it apart from other species within the genus. However, the abdominal spots of this species may sometimes be indistinct and appear blurry. In tergites III to V, these spots may be partially obscured by brown spots, but tergite II consistently displays four clearly visible elliptical spots. The overall shape of this new species is similar to that of *M.kibunensis*, and it shares the same gonostylus morphology. However, *M.kibunensis* lacks an inferior volsella and anal point, while this new species possesses a protrusion on the inferior volsella. The shape of the inferior volsella is comparable to that of *M.excavata* sp. nov., but this new species can still be differentiated by the presence of color spots on the tergites, the presence of the anal point, and a higher HR value (1.91–2.09) compared to *M.excavata* sp. nov. Based on COI sequences, the pairwise distances between *M.quadrimacula* and *M.kibunensis*, and between *M.quadrimacula* and *M.excavata*, are 0.119–0.125 and 0.131, respectively, further setting it apart from them.

#### Distribution.

Hubei Province, Oriental China.

### ﻿Key to adult males of genus *Macropelopia* from China

**Table:** 

1	Wings with distinct color spots	**2**
–	Wings without color spots; only dark markings on cross-vein r-m	**M. (M.) notata (Meigen)**
2	Tergites with brown spots	**3**
–	Tergites without brown spots	**6**
3	Foretibial comb with 9–15 small bristles, inferior volsella significantly larger	**M. (M.) paranebulosa (Fittkau)**
–	Foretibial comb with 5–7 small bristles, inferior volsella absent or undeveloped	**4**
4	Tergites IX is uncovered gonocoxite, base of the anal point is wide, presenting a triangular anal point; posterior setae on tergite IX are mainly distributed in the central part	**M. (M.) quadrimacula Xu & Fu, sp. nov.**
–	Tergites IX covered parts of gonocoxite, anal point absent; posterior setae on tergite IX are mainly distributed at the posterior margin	**5**
5	Coxa, trochanter, part femur and apex of tibia dark brown, and apex of femur pale; antennal ratio 1.7–1.9; whole wing with dense setae on membrane	**M. (M.) kibunensis (Tokunaga)**
–	Coxa, trochanter, femur and apex of tibia dark brown; antennal ratio 2.24; upper part of the wings with dense setae on membrane, but they are significantly reduced below the CU vein and M2+3 vein	**M. (M.) excavata Xu & Fu, sp. nov.**
6	Tergites pale brown, foretibial comb with 11–15 small bristles, and anal point absent	**M. (M.) pergrandis (Tang & Niitsuma)**
–	Tergites all brown, foretibial comb with 6 bristles and anal point present	**M. (M.) decedens (Walker)**

## ﻿Discussion

Based on the statistical data presented in this study, it has been revealed that there are currently seven species of the genus *Macropelopia* known to be distributed in China. However, it is important to note that the species Macropelopia (M.) notata and M. (M.) decedens, as reported by Wang et al. (2020), lack sufficient morphological characteristics for definitive identification. While we have included these species in the key provided in this study to reflect the current research records, further investigations are necessary to verify their distribution with more certainty.

**Figure 3.**
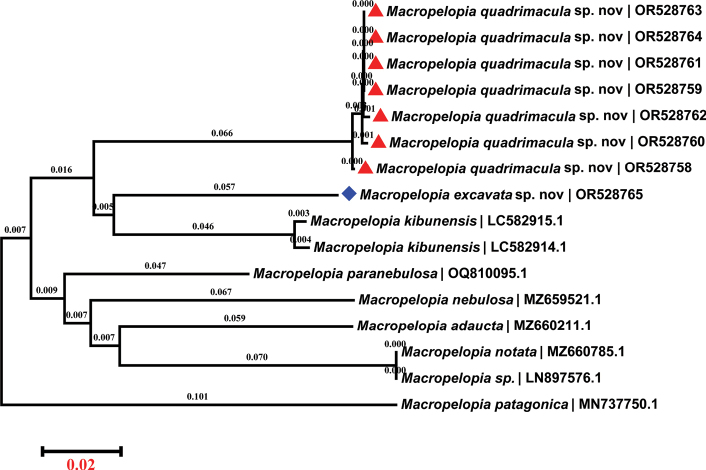
Neighbor-joining tree based on cytochrome *c* oxidase subunit I (COI) of sixteen *Macropelopia* specimens. Numbers on branches refer to the evolutionary distances computed using the Kimura 2–parameter method (Kimura 1980) and represent number of base substitutions per site. Taxa names include scientific names and GenBank accession numbers of corresponding COI gene fragments.

We successfully obtained eight COI sequences for two new species and downloaded an additional eight sequences for seven species from the National Center for Biotechnology Information (NCBI). Their taxonomic names and GenBank accession numbers can be found in Fig. 3. By utilizing the neighbor-joining method (Tamura et al. 2021) for constructing a phylogenetic tree, our analysis revealed that *M.excavata* sp. nov. and *M.kibunensis* are closely related, as they appeared on the same branch of the tree. This finding is consistent with their shared morphological characteristics, such as the similar coloration of the tergites, anal point absent, and thorax features. *Macropelopiaquadrimacula* sp. nov. is shown to be the sister group to (*M.excavata* sp. nov. + *M.kibunensis*); the primary distinguishing features between *M.quadrimacula* sp. nov. and *M.excavata* sp. nov. lie in their hypopygium and the presence or absence of a dorsal stripe on their tergites. These results exemplify a strong congruence between the molecular and morphological data. Interestingly, our findings contrast with the description of *M.kibunensis*, which includes yellow femora and the wing with dense setae. In contrast, the two new species possess brown femora and a dorsal stripe on the tergites. Thus, within the genus *Macropelopia*, key criteria for morphological classification encompass the characteristics of the hypopygium and dorsal stripe patterns on the tergites, followed by the markings and macrotrichia of the wing, and the color and features of the legs.

Chironomids offer the advantage of having three distinct stages (larvae, pupae, and adults); the morphology of the larvae and pupae of *Macropelopia* also plays an important role in species delineation (Fittkau 1962, Roback 1978, Fittkau and Murray 1986, Tang and Niitsuma 2020). This study primarily focuses on morphological differences in the adult stage; collecting specimens from multiple life stages simultaneously remains a challenge. However,previous studies have demonstrated that COI is suitable for summarizing sequence diversity and detecting taxonomically challenging species within *Macropelopia* (Silva and Pinho 2020). Therefore, we anticipate further studies on species delineation using the COI gene segment to enhance the reliability of new species establishment. The analysis of partial DNA barcode sequences supports *Macropelopiaexcavata* sp. nov. and *Macropelopiaquadrimacula* sp. nov. as valid species.

## Supplementary Material

XML Treatment for Macropelopia

XML Treatment for Macropelopia excavata

XML Treatment for Macropelopia quadrimacula

## ﻿Additional information

### Author ORCIDs

Yue Fu https://orcid.org/0000-0002-4045-4352

### Data availability

All of the data that support the findings of this study are available in the main text.
